# A Review of Metallothionein Isoforms and their Role in Pathophysiology

**DOI:** 10.1186/1477-7819-9-54

**Published:** 2011-05-20

**Authors:** N Thirumoorthy, A Shyam Sunder, KT Manisenthil Kumar, M Senthil kumar, GNK Ganesh, Malay Chatterjee

**Affiliations:** 1 Dept. of Pharmaceutics, Cherraan's College of Pharmacy, 521 Siruvani Road, Perur, Coimbatore-39, TN, India; 2 Dept. of Pharmacology, College of Pharmacy and Nursing, University of Nizwa, Sultanate of Oman; 3 Dept. of Pharmacology, K.M.C.H. College of Pharmacy, Kovai Estate, Kalapatti Road, Coimbatore -35, TN, India; 4 Dept. of Pharmaceutics, JSS College of Pharmacy, Ooty, TN, India; 5 Dept. of Pharmaceutical Technology, Jadavpur University, Kolkatta. WB, India

**Keywords:** Metallothionein Isoforms, MT-I, MT-II, MT-III, MT-IV

## Abstract

The Metallothionein (MT) is a protein which has several interesting biological effects and has been demonstrated increase focus on the role of MT in various biological systems in the past three decades. The studies on the role of MT were limited with few areas like apoptosis and antioxidants in selected organs even fifty years after its discovery. Now acknowledge the exploration of various isoforms of MT such as MT-I, MT-II, MT-III and MT-IV and other isoforms in various biological systems.

Strong evidence exists that MT modulates complex diseases and the immune system in the body but the primary function of MT still remains unknown. This review's main objective is to explore the capability to specifically manipulate MT levels in cells and in animals to provide answers regarding how MT could impact those complex disease scenarios.

The experimental result mentioned in this review related among MT, zinc, cadmium, diabetic, heart disease, bone retardation, neuro toxicity, kidney dysfunction, cancer, and brain suggest novel method for exploration and contribute significantly to the growing scientist to research further in this field.

## Introduction

The metallothionein (MT) was first isolated in 1957 from the cortex of horse kidney as a cadmium binding protein [1]. This protein was first reported by Kagi and Vallee in 1960 and by Kojima in 1976 as cysteine-rich (33 mol %), low molecular weight (7 kDa), heat-stable and metal binding protein.

There are at least ten known closely related metallothionein proteins expressed in the human body. In humans, large quantities are synthesized primarily in the liver and kidneys, however they have been found at a number of other sites as well. Its production is dependent on availability of the dietary minerals zinc and selenium, and the amino acids histidine and cysteine present in the body.

This protein has properties like detoxification of heavy metals like mercury and cadmium, homeostasis of essential metals including copper and zinc, antioxidation against reactive oxygen species, protect against DNA damage, oxidative stress, cell survival, angiogenesis, apoptosis, as well as increase proliferation, etc in the body [2]. In general the MT is known to modulate three fundamental processes: 1) the release of gaseous mediators such as hydroxyl radical or nitric oxide; 2) apoptosis, and 3) the binding and exchange of heavy metals such as zinc, cadmium or copper.

### MT and Its Isoforms

MT isoforms are classified based on various factors like molecular weight, metal which bind, encoded genes, chromosomes, binding atoms, amino acids environment etc. Broadly it is classified as major and minor groups. The major groups are MT-1 and MT-2; these are the unique structure which is identical for the two major isoforms binds 7 g atoms of divalent metals like zinc and cadmium. The MT-3 and MT-4 are minor isoforms which are normally found in specialized cells. The MT-3 protein was first isolated as a growth inhibiting factor (GIF) from brain neurons, and the MT-4 protein was found in stratified epithelium [3].

In human, the MT proteins are encoded by a family of genes which are located on chromosome 16q13, and may involve at least 10 identified functional genes [3]. This main isomers are again subdivided as MT I a, MT I b, MT I c etc or any other resemble pattern.

Another study classified MT I and II based on the sequences and that do not consider either the state of the thiols or the cargo, namely which metal is associated with these protein but there is a scarcity of functional studies that would relate them to class I MTs.

The four isoforms of MT are identified in mammals, three of which, MT-I, II and III are found in the central nervous system. The expression of MT-I/II is mainly localized in glias and is induced by exposure to metals including Hg, Cd, Cu and Zn, cytokines and ROS. On the other hand, the MT-III isoform are mainly present in neurons and are not easily induced by exposure to the above agents [4].

Four classes of MTs have been characterized in mammals. The MT-I and II genes are expressed in many tissues, and at a particularly high level in liver and kidney. Expression of MT-III is restricted to the brain and to male reproductive organs, while that of MT-IV is specific to stratified squamous epithelia, since mice that cannot synthesize either MT-I or MT-II and reproduce normally. Mice lacking MT-III do not reveal any neurological or behavioral deficiencies [5].

A another classification based on a large family of eukaryote and some are in prokaryote refers to three classes: (i) proteins with sequences related to mammalian MT, (ii) proteins with sequences not related to mammalian MT, and (iii) peptides that are not genetically encoded [6]. All above classification are listed in below table 1.

**Table 1:** Classification of MT-Isomers

S.No	Based on		Classification
1.	Metals Present		Major -MT-1 &MT-2
			Minor - MT-3 &MT-4

2.	Proteins are encoded by a family of genes which are located on chromosome 16q13		MT I a, MT I b, MT I c etc

3.	Biological system	Central nervous system	MT-I, MT-II and MT-III
		Neurons	MT-III

4.	Family of eukaryote and some are in prokaryote	Proteins with sequences related to mammalian MT	MT- i
		Proteins with sequences not related to mammalian MT	MT- ii
		Peptides that are not genetically encoded	MT- iii

5.	Expressed in tissues	Liver and kidney	MT-I and MT-II
		Brain and to male reproductive organs	MT-III
		Specific to stratified squamous epithelia	MT-IV

### General task of MT isoforms in the Body

Four primary MT proteins and their important role in the body

• MT-I and II are present in all cells throughout the body. They regulate copper and zinc, are involved in cell transcription, detoxify heavy metals, play a role in immune function, and are involved in a variety of G.I. tract functions.

• MT-III is found primarily in the brain and functions as a growth inhibitory factor in the brain. MT-III is located primarily in the central nervous system with small amounts present in the pancreas and intestines. It plays a major role in the development, organization and programmed death of brain cells.

• MT-IV is found in the skin and upper G.I. tract. They help regulate stomach acid pH, taste and texture discrimination of the tongue and help protect against sunburn and other skin traumas.

## Structure of MT

### Spectroscopic Characterization

Few studies have revealed the structure and characterization of MT. The three-dimensional protein structure of this was reported by both X-ray crystallography and NMR spectroscopy in the 1990s. Structural studies have shown that this unusual protein with 61 amino acids (mammalian MT) can bind with both essential metals (zinc and copper) and toxic metals (cadmium and mercury) in two distinct cluster structures within the molecule. One cluster is closer to the N-terminal and three metal atoms are bound to nine cysteines with three bridging sulfur atoms, while in the second cluster closer to the C-terminal and four metal atoms are bound to 11 cysteines with five bridging sulfur atoms [7].

### Fluorescent probes for the structure of MT

Fluorescence methods for demonstrate the structure of human metallothionein in vivo which depends on the presence of metal ions and the redox environment of amino acids. Attachment of fluorescent labels generated metallothionein FRET (fluorescence resonance energy transfer) sensors for investigating its structure and function in living cells differential chemical modifications of its cysteine thiols with fluorescent probes allowed three states in structure: 1)Metallothionein (zinc-bound thiolate), 2) Thionein (free thiols), and 3) Thionin (disulfides). Interrogation of this zinc-binding property with fluorescent chelating agents revealed that the affinities of the seven zinc ions over four orders of magnitude [6].

## MT Isomers and Cancer

### In Breast Cancer

These isoforms are expressed in a tissue specific pattern and may play distinct roles in the different cell types. There are several reports on the expression of certain specific isoforms in various human tumors. The mRNA of MT-1 series named as A, E, F, G, H, X and MT-3 isoforms but not MT-1B and MT-4 isoforms have been detected in breast cancer tissues. The MT-2A mRNA transcript which has been reported to be highest among all the functional isoforms detected in breast tissues and is positively correlated with cell proliferation and histological grade. Expression of MT-1F isoform has also been found to influence histological differentiation in invasive breast cancer since estrogen is known to play important role in breast cancer tumorgenesis, the MT-1E isoform has been postulated to participate in alternative processes that replace the function of estrogen. It has also been reported that MT-3 isoform overexpression is associated with a poor prognosis for patients with breast cancer [3].

### In Renal Tumor

The renal cell cancer tissue shows three different type of expression as up-regulation of MT-2A, down-regulation of MT-1A and MT-1G transcripts. Expression of the MT-3 isoform has been reported in the tubules of normal kidney and also in renal cell carcinoma along with other isoforms of MT. The expression of the MT-3 isoform in cancerous bladder tissues which was absent in normal bladder tissues, and suggested its use as a potential biomarker for bladder cancer. They have also shown high levels of MT-1X mRNA expression in bladder cancer. The MT-3 isoform which was originally reported as specific to brain has been demonstrated in normal human kidney, renal carcinoma, bladder cancer and prostatic adenocarcinoma [3].

### In Prostate Cancer

In normal prostate tissue, the MT-I A, E, X and MT-2A isoforms were present but there was a down-regulation of the MT-IX isoform in advanced prostate cancer. It was reported that MT-1 and MT-2 isoforms may be related to the proliferative activity of breast, colon and prostate human cancers [3].

### In Papillary Thyroid Cancer

MT isoforms have not been much studied in papillary thyroid cancer. The function of MT1 and MT2 isoforms in papillary thyroid cancer cells (KAT5) demonstrated that KAT5 cells expressed eight functional MT1 and MT2 isoforms induced by cadmium. Elevated calcium and activated ERK1/2 predated MT expression. The alternation in cell cycle disappeared when the expression of MT isoforms was blocked by calcium inhibitor or ERK1/2 inhibitor. Collectively, KAT5 cells express eight functional MT1 and MT2 isoforms in a pathway controlled by calcium and ERK1/2. The elevation of the MT isoforms contributes to the decreased G0/G1 but increased G2-M phase revealed a novel pathway for the expression of the functional MT in papillary thyroid cancer. Bone thyroid cancers are classified as papillary, follicular, medullary, and undifferentiated or anaplastic [8]. Table 2 is to express the role of MT isoforms on cancer.

**Table 2:** Showing the Isoforms of MT on Tumorgenesis

S.NO	CANCER	ISOFORMS	ROLE
1	Breast Cancer	MT-1 A,E,F,X, H,G, MT-3, MT-2A.	1) MT-1E: Alternative processes that replace the function of estrogen i;e breast cancer tumorgenesis. 2) MT-3: Poor prognosis for patients with breast cancer.

			3) MT-2A: Detected in breast tissue is positively correlated with cell proliferation and histological grade. 4) MT-1F: It found to influence histological differentiation in invasive breast cancer.

2	Renal Cancer	MT-1A, MT-1G, MT-IX, MT-3.	1) MT-3: Normal kidney & also in renal cell carcinoma, cancerous bladder tissues,(which was absent in normal bladder tissues)prostatic adenocarcinoma.. 2) MT-IX:Bladder cancer.

3	Prostate Cancer	MT-1A, MT-1E, MT-1X, MT-2A, MT-1, MT-2.	1) MT-IX:Advanced prostate cancer. 2) MT-1, MT-2: proliferative activity of breast, colon & prostate human cancer.

4	Papillary Thyroid Cancer	MT-1, MT-2.	Thyroid Cancer cells (KAT5 cells) + Calcium elevated ERK1/2 → 8 functional MT-1&MT2 induced By cd → MT expression+ Decrease in G0, G1 phase, Increase in G2-M phase → Novel pathway for Functional MT expression.

## Bone Growth Retardation and MT Isomers

Bone growth retardation, zinc and its binding protein MT are important in regulating growth and development of bone. A study on relationship between dietary Zn and MT interact in regulating bone growth were reported that the MT mice, having lower Zn concentrations in plasma and long bone, showed growth retardation as demonstrated by lower body length gain, shorter and smaller tibia/femur, lower chondrocyte proliferation, reduced metaphysis heights, but increased osteoclast densities on trabecular bone, particularly in mice fed Zn low diet (Zn-L). The mRNA expression of MT-I&II was induced in mice fed with the Zn-L diet possibly compensating for Zn limitation that interact between dietary Zn and endogenous MT is important for maximal bone growth, and particularly important in the regulation of Zn pool for bone growth during moderate Zn limitation [9].

## Role in Oxidative Stress

Recent experiments have shown that thiolate ligands in MT confer redox activity on zinc clusters. This strongly suggests that MT would control the cellular zinc distribution as a function of the cellular energy state [2]. A review of report have proved that the anti oxidant property of MT enhances in presence of zinc. The zinc redox-dependent functions of MT are important for the regulation of physiological processes that depend on zinc and the pathological processes in which oxidative stress mobilizes zinc. The decrease of zinc availability from MT suggests that the mutant MT is either less reactive towards nitric oxide or it is in an oxidized state and does not bind sufficient amounts of zinc [6].

A DNA microarray used to examine genes induced by gallium nitrate in specified cells named as CCRF-CEM cells. This study found that gallium increased MT-2A and heme oxygenase-1 (HO-1) gene expression and altered the levels of other stress-related genes. Gallium nitrate increased the phosphorylation of p38 mitogen-activated protein kinase and activated Nrf-2, a regulator of HO-1 gene transcription. Gallium induced Nrf-2 activation and HO-1 expression were diminished by a p38 MAP kinase inhibitor. This concludes that gallium nitrate induces cellular oxidative stress that triggers the expression of HO-1 and MT2A through different pathways [10].

### Protection in cadmium toxicity

Cadmium (Cd) is an environmental pollutant ranked eighth in the top 20 hazardous substances and the human activity has markedly increased the distribution of Cd in the global environment. Cd is toxic to number of tissues in body. Prolonged exposure to Cd produces nephrotoxicity, osteotoxicity, and immunotoxicity. this is also classified as a human carcinogen causing genitourinary disorders like tumors of the lung, prostate, injection site, and other tissues. Most of Cd in the body is bound to a small, cysteine-rich, metal binding protein MT [11].

This protein expression in Cd-induced tumors varies depending on the type and the stage of tumor development. High levels of MT are detected in Cd-induced sarcomas at the injection site and sarcomas metastases are devoid of MT suggest the critical role for protecting human health from Cd toxicity either by neither detoxification nor heavy metal binding [12].

## MT in Kidney

The prevalence of cadmium-related kidney dysfunction among population groups residing in cadmium contaminated areas in China report reveal a dose-response relationships between urinary-Cd and renal tubular dysfunction such as urinary beta-2-microglobulin or N-acetyl-beta-D-glucosaminidase-NAG or urinary albumin, a biomarker of glomerular kidney dysfunction. Since long term cadmium exposure in occupational and general environments may give rise to kidney dysfunction. These dose-response relationships include:

1) MT- mRNA levels in peripheral blood lymphocytes,

2) Biomarker of the ability of each person, and

3) To synthesize metallothionein (a protein known to provide intracellular protection against cadmium toxicity [13].

## In The Central Nervous System

The transgenic models of MT expression were established using various experimental approaches. This important protein plays a major role in the defense against neurodegenerative disorders and other injuries, influence tissue architecture and cognition, finally protect against mercury neurotoxicity [14].

Nicotine treatment, which can improve working memory, eliminated the impairment associated with the deletion of the MT-1 and MT-2 genes in a dose-related fashion after acquisition training in the aging adult mice. These have been suggested its roles in metal physiology or cellular protection are involved in spatial learning and memory function. These studies conclude that MT has important functions in the central nervous system and brain because MT-1 and MT-2 protect the central nervous system from damage induced by interleukin, 6-aminonicotinamide, kainic acid, and physical injury [15].

This investigates also studied that, transition metals have been associated with impaired neurological development, and neurobehavioral activity. The role of MT in learning and memory of mice with deletions of two metallothionein genes (MT-1 and MT-2) were trained on a win-shift task in an 8-arm radial maze. The parental strain of these mice learned the maze at a normal rate over an 18-session acquisition period. In contrast, the MT-1/MT-2-null mice, which had a similar choice accuracy level at the beginning of training, showed a poorer rate of learning during the training period. In addition, the MT-1/MT-2-null mice showed significantly less choice of accuracy than the parental strain [15].

## MT in Heart Disease

### MT-IIA in heart-derived cell line in human confers oxidative protection

MT is a metal binding protein and cardio protective. In order to understand the molecular mechanisms underlying the role of MT in the heart a study established a stable MT-IIA over-expressing cardiac cell line, and evaluated its anti-oxidative property. The transfected cell line (H9c2MT7) exhibited similar growth kinetics and morphology.

The western blotting analysis of this study showed that H9c2MT7 had a remarkable increase in MT protein level compared with the parent cell line H9c2. Upon addition of 25 M ZnSO_4 _showed an undetectable effect on the induction of endogenous MT, but it likely stabilized the MT protein that is expressed only in H9c2MT7 cells. In addition, transfection of MT conferred cellular resistance to cadmium toxicity have established a stable human MT-IIA over-expressing cardiac cell line; and this cell line showed a markedly increased oxidative protection and would be useful for dissection of the mechanisms of MT in the cardiac protection [16].

## MT and Diabetes

### Apoptosis and Pathological Remodeling in the Diabetic Heart

A preclinical research in 2008 concluded the result that the acute angiotensin II administration to WT mice or neonatal cardiomyocytes increased cardiac apoptosis, nitrosative damage, and membrane translocation of the nicotinamide adenine dinucleotide phosphate oxidase (NOX) isoform p47phox. Prolonged administration of suppressor doses of Ang II (0.5 mg/kg every other day for 2 weeks) also induced apoptosis and nitrosative damage in both diabetic and non-diabetic WT hearts, but not in diabetic and non-diabetic MT-TG hearts. Long-term follow-up (1 to 6 months) of both WT and MT-TG mice after discontinuing Ang II administration revealed progressive myocardial fibrosis, hypertrophy, and dysfunction in WT mice but not in MT-TG mice.

**Figure 1:**
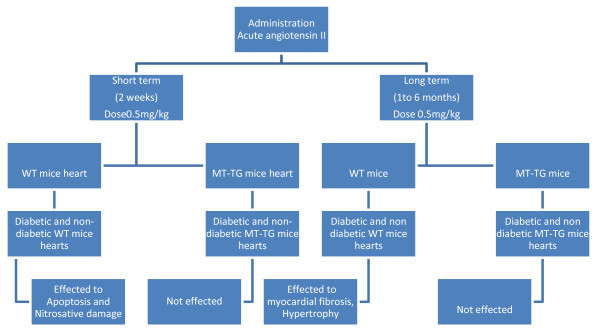
**A Chart Explain a study on Apoptosis and Pathological Remodeling In the Diabetic Heart**.

This study finalize MT suppresses Ang II-induced NOX-dependent nitrosative damage and cell death in both non-diabetic and a diabetic heart early in the time course of injury and prevent the late development of Ang II-induced cardiomyopathy and the same has been expressed in the figure 1[17].

### MT, Zinc and Diabetes

Diabetes and polymorphisms in human genes control the cellular availability of zinc ions. One protein is the zinc transporter ZnT-8 that supplies pancreatic b-cells with zinc. The other is MT 1A, a member of a protein family that links zinc and redox metabolism. Changes in the availability of zinc ions modulate insulin signaling and redox processes. Both zinc and MT protect cells against the redox stress that occurs in diabetes and contributes to its progression towards diabetic complications, including heart disease.

### Zinc an insulinomimetic

**Figure 2:**
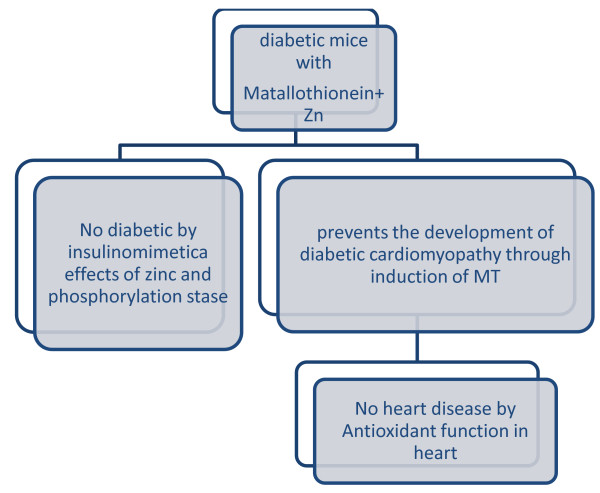
**Mechanisms of MT and Zinc as an insulinomimetic in mice**.

Figure 2 shows that, the MT in presence of zinc can able to reduce diabetic by insulinmimetic activity through phosphorylation thereby diabetic induced heart disease are also controlled.

In diabetes, these functions of MT come to bear on insulin signaling and coronary heart disease. Insulin and zinc ions have potent stimulatory effects on lipogenesis and glucose uptake. Zinc-deficient animals are less sensitive to insulin. Zinc can replace insulin in mammalian cells cultured in serum-free media [18]. The actions of zinc are intracellular because zinc increases the phosphorylation state of the insulin receptor, and hence, protein phosphorylation downstream in the insulin signaling pathways [19]. It has been suggested that zinc inhibition of protein tyrosine phosphatase 1B, the major phosphatase controlling the phosphorylation state of the insulin receptor, is responsible for these insulinomimetic effects of zinc [20]. Coronary heart disease is the leading cause of mortality in type 2 diabetes. The diabetics with are C-allele carriers are more likely to develop cardiovascular complications might indicate a role for MT and zinc in the pathogenesis of diabetic heart disease. In mice, zinc supplementation prevents the development of diabetic cardiomyopathy through induction of MT, which has an antioxidant function in the heart [21].

Low serum zinc in type 2 diabetics is significantly correlated with mortality from coronary heart disease [22]. Despite the presence of extensive zincuria in type 2 diabetics, there is still no consensus about whether or not the zinc in their blood plasma reflects a generally reduced zinc status [23]. Also, a recent review of the literature concludes that there is no evidence of zinc supplementation being effective in preventing diabetes [24].

Numerous studies have been proved the antidiabetogenic properties of zinc supplementation in both diabetic laboratory animals and in humans. The above findings indicate that differences in the cellular availability of zinc in both insulin producing b-cells and in insulin target cells are associated with risk for diabetes in specific human populations [25,26].

## Discussion

The past decades have demonstrated increased focus on its isoforms like MT-I, MT-II, MT-III and MT-IV. MT-I and II were mainly focused in oncogenesis, tumor progression, therapy response, and patient prognosis. Studies have reported increased expression of MT-I and II mRNA and protein in various human tumors; such as breast, kidney, lung, nasopharynx, ovary, prostate, salivary gland, testes, urinary bladder, cervical, endometrial, skin, and pancreatic cancers, as well as in melanoma and all, where in some cases MT-I and II expression correlates with tumor grade/stage, chemotherapy/radiation resistance, and poor prognosis.

However, MT is down-regulated in certain tumors such as hepatocellular carcinoma and liver adenocarcinoma. Hence, the expression of MT is not universal to all human tumors, but may depend on the differentiation status and proliferative index of tumors, along with other tissue factors and gene mutations. In certain tumors such as germ cell carcinoma, the expression of MT is closely related to the tumor grade and proliferative activity [3]. All this studies confirm the direct or indirect link between MT isoforms and tumor prognosis.

The expression of MT-I and MT-II (MT-I/II) isoforms were measured together with Western blotting, copper level, and lipid peroxides amounts increased in an age-dependent manner in the spinal cord, the region responsible for motor paralysis concluded, MTs could have a disease modifying property [4].

The finding on kidney correlates MT as a biomarker of glomerular kidney dysfunction by dose-response relationships between urinary-Cd and the prevalence of increased levels of biomarkers in urine of renal tubular dysfunction such as urinary beta-2-microglobulin or N-acetyl-beta-D-glucosaminidase - NAG or urinary albumin [13].

The relationships among MT, zinc, and oxidative stress suggest many new areas for exploration, with the expectation that results forthcoming from experiments designed on the basis of these new findings will contribute significantly to our understanding of the role of zinc in diabetes and to the prevention and treatment of diabetes and its complications in susceptible populations [6]

## Conclusion

Commonly it is understood that, MT is an endogenous substance which regulates metal level in animal and human body. The MT has got fingers like arrangement and when metal level is elevated in the body these fingers are triggered to bind with them. If metal level is low in the body, it becomes vice- versa. All together the expression pattern for MT will be a changed in the body when any diseases caused due to metal factor.

This review article conclude that, many independent groups of investigators found direct casual relationships between MT and pathophysiology but more pronounced reasons among those was endogenous and exogenous stimuli including glucocorticoids, interferon, interleukin-1, progesterone, vitamin D_3 _endotoxins, serum factors, heavy metals, storage of metal ions and regulation of cellular zinc etc. may trigger the expression of MT in human and animal's body.

However more research is required to test the important hypothesis of either MT could consider as biomarker, alter toxicity and susceptibility of humans.
